# The Effect of Acidic pH on Microleakage of Mineral Trioxide Aggregate and Calcium-Enriched Mixture Apical Plugs

**Published:** 2014-10-07

**Authors:** Hossein Mirhadi, Fariborz Moazzami, Sareh Safarzade

**Affiliations:** a *Department of Endodontics, Dental School, Shiraz University of Medical Sciences, Shiraz, Iran;*; b *Student Research Committee, Dental School, Shiraz University of Medical Sciences, Shiraz, Iran*

**Keywords:** Acidic pH, Apical Plug, Calcium-Enriched Mixture, CEM Cement, Fluid Filtration, Microleakage, Mineral Trioxide Aggregate, Sealing Ability

## Abstract

**Introduction:** The purpose of this laboratory study was to evaluate the effect of acidic pH on the sealing ability of calcium-enriched mixture (CEM) cement and mineral trioxide aggregate (MTA) apical plugs. **Methods and Materials:** Seventy single-rooted human maxillary anterior teeth were recruited. The teeth were randomly divided into 4 experimental groups (*n*=15), and 1 negative and 1 positive control groups of 5. The root canals were cleaned and shaped and the terminal 3 mm of the roots were resected. Then MTA and CEM cement plugs were condensed in apical region with 3 mm thicknesses. The samples were exposed to pH values of 5.5 and 7.4. Leakage was evaluated by the fluid filtration technique at 1, 7, 14, 30 day intervals. Data were analyzed by the repeated measures MANOVA, one-way ANOVA and MANOVA/Bonferroni test. **Results: **Acidic pH significantly reduced the sealing ability of MTA after 1, 14 and 30 days (*P*<0.05). The rate of microleakage in CEM cement samples in acidic pH was significantly greater than that in neutral pH in day 30 (*P*<0.05). There was no significant difference between the sealing property of MTA and CEM cement at both pH levels (*P*>0.05). **Conclusion: **It can be concluded that the CEM cement exhibited similar sealing ability as MTA at both pH levels. In addition, an acidic pH environment reduced the sealing ability of MTA and CEM cement after 30 days.

## Introduction

Most of the endodontic failures occur as a result of egression of intracanal irritants into the periapical tissues [1]. An ideal orthograde or retrograde filling material should prevent leakage of microorganisms and their byproducts [2]. On the other hand, it is usually challenging to achieve an adequate apical stop in teeth with incomplete root development or apical root resorption. Regular orthograde root-filling and sealing of these teeth with open apices is hard if not impossible; as a result, placement of an apical plug to achieve an adequate apical seal is suggested in such cases [3-5]. Mineral trioxide aggregate (MTA) and calcium-enriched mixture (CEM) cement have been advocated as osteo-conductive apical plugs [6, 7]. MTA has good sealing ability, sets in the presence of blood, and is biocompatible and all these properties make it a good candidate for apical plug [8]. However, it encompasses some drawbacks such as extended setting time, poor handling, and high cost [9]. On the other hand CEM cement, apart from its good sealing ability and favorable biologic response, has more flow, less film thickness [10, 11] and shorter setting time (less than 1 h) compared to MTA [10, 11]. Asgary *et al*. [10] showed that CEM cement had significantly more antibacterial properties than MTA. CEM cement has been used for the repair of furcal perforation, pulp cap and pulpotomy treatments, similar to MTA [12].

In certain clinical situations such as presence of infection, the periradicular area has an acidic pH which can potentially affect the properties of freshly applied apical plugs. It has been reported that a low pH environment can reduce the strength and hardness of MTA, inhibit the setting reactions and increase the solubility of test materials [13, 14, 15]. Saghiri *et al.* [16] demonstrated that acidic pH caused the leakage of MTA in shorter time duration compared to neutral pH.

To date no investigation has evaluated the effect of an acidic pH on sealing ability of CEM cement apical plug. Hence, the aim of this laboratory study was to assess effect of acidic pH on the microleakage of MTA and CEM cement apical plugs by means of fluid filtration technique.

## Methods and Materials

Seventy human maxillary anterior teeth with single straight roots and mature apices were recruited for this study. The samples were stored in 1% sodium hypochlorite (NaOCl) for 48 h and then restored in normal saline solution until the start of procedure. Teeth with cracks and calcified canals were excluded.

The crown of each tooth was removed using a fissure bur (010. Maillefer, Ballaigues, Switzerland) installed on a high-speed handpiece to equalize the length of teeth. The teeth were randomly divided into 2 experimental groups of 30 for Angelus MTA (Angelus, Londrina, Paraná, Brazil) and CEM cement (BioniqueDent, Tehran, Iran); then each group was further divided into two subgroups (*n*=15) for neutral and acidic pH. In addition, two negative and positive control groups, each comprising of 5 samples, were considered. The working length (WL) of each canal was determined by placing a #15 K-file (Maillefer, Ballaigues, Switzerland) in the canal until it appeared from the apical foramen. The WL was established 1 mm shorter than this length. The root canals were cleaned and shaped using ProTaper rotary system (Dentsply Maillefer, Ballaigues, Switzerland) to size F3 according to the manufacturer’s protocol. During instrumentation, after each rotary file two mL of 2.5% NaOCl was used.

Then the apical 3 mm of the roots were resected. The apical region of teeth was dilated with piezo drills (Dentsply, Maillefer, Ballaigues, Switzerland) up to size 3 to simulate open apex teeth. The instrumented canals were dried with paper points (VDW, Munich, Germany). Then MTA and CEM cement were prepared according to their manufacturers’ instructions. The cements were condensed to the apical end with # 3 and 4 hand pluggers (Dentsply, Maillefer, Ballaigues, Switzerland) with a rubber stop placed 3 mm shorter than the WL. In the experimental groups, the thickness of the apical barrier was confirmed by radiographs. The entire root surface was coated with two layers of nail varnish, except for the area corresponding to the resected root-end surface.

In the positive control group, no apical plug was used. In the negative control group, a 3-mm MTA apical plug was placed and then the entire root surfaces and the surface of MTA was covered by two coats of nail varnish.

To provide acidic pH environment, butyric acid (BA) with pH of 5.5 was employed [17]. In acidic pH group, the tooth was placed in pieces of gauze soaked in BA for 3 days [16]. The gauze was refreshed every day to maintain sufficient acidic environment. The samples of the neutral pH group were placed in the pieces of gauze soaked in synthetic tissue fluid (STF) with neutral pH (containing 1.7 g of KH_2_PO_4_, 11.8 g of Na_2_HPO_4_, 80.0 g of NaCl and 2.0 g of KCl in 10 L H_2_O), for 3 days [16].

Microleakage was evaluated by the fluid filtration technique with 20 cmH_2_O pressure [18]. In this method, a device was prepared by attaching two micropipettes perpendicular to each other.

The teeth were fixed at the end of a horizontal micropipette and 20 cm normal saline was poured in the vertical micropipette, simulating the pressure of 20 cmH_2_O. Microleakage for each tooth was measured in 1, 7, 14, 30-day intervals. Then, the bubble displacement of each interval (0-1, 1-7, 7-14 and 14-30 days) was reported. The rate of microleakage (RML) of each interval was calculated and expressed in µL/min [19].

Data were analyzed by the repeated measures MANOVA test to evaluate the changes in sealing ability over time. The one-way ANOVA/LSD was used among group analyses to compare the mean of RML among the 4 groups at each time. In addition, repeated measures MANOVA/Bonferroni test was used to compare RML within the groups. The level of significance was set at 0.05 for all tests.

## Results

The bubble did not displace in the micropipette in negative control group, while in the positive control group the fluid flow was observed as soon as the pipette was opened. All experimental groups demonstrated various amounts of microleakage (Table 1).

There was a significant interaction effect between different time intervals and 4 groups. Therefore, we used one-way ANOVA/LSD as a subgroup analysis to compare the four groups’ mean RML at each time interval.

**Figure 1:**
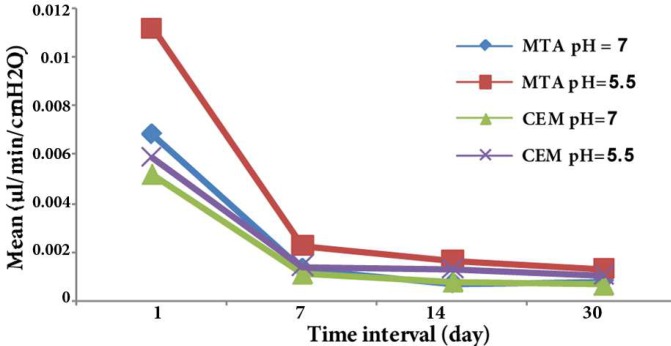
Mean microleakage values of MTA and CEM cement apical plugs at various pH levels and time intervals

The RML of MTA in an acidic environment (pH=5.5) was significantly greater than that in the neutral pH (*P*<0.05), except in day 7 (*P*>0.05). An acidic pH of 5.5 did not significantly affect the RML of CEM cement in 1, 7, and 14 days. In day 30, the RML of CEM significantly increased in an acidic pH compared with a neutral pH. There was no statistically significant difference at acidic pH in all time intervals (*P*>0.05). These materials also exhibited similar sealing ability in neutral pH. The RML was reduced over time and it was more detectable in the second interval (Figure 1).

**Table 1:** Mean (SD) of microleakage values (µL/min/cmH2O ×10^-^³) for MTA and CEM cement as apical plugs in different time intervals

	**pH Level**	**Mean (SD)**				
		**Day 1**	**Day 7**	**Day 14**	**Day 30**	**N**
**MTA**	**Acidic**	11.1 (5.8)	2.2 (1.7)	1.6 (0.7)	1.3 (0.4)	15
	**Neutral**	6.8 (4.7)	1.3 (0.5)	0.7 (0.3)	0.8 (0.3)	15
**CEM**	**Acidic**	5.8 (2.6)	1.4 (0.8)	1.3 (0.6)	1 (0.4)	15
	**Neutral**	5.1 (3.1)	1.1 (0.8)	0.8 (0.3)	0.6 (0.3)	15

## Discussion

This study compared the sealing ability of MTA and CEM cement as apical plugs, in acidic environment. Acidic environment reduced the sealing ability of MTA or CEM cement after 30 days without any significant differences between the two biomaterials.

In certain clinical situations, MTA or CEM cement might be used in the presence of inflammation. The pH of a human abscess has been reported to be as low as 5.0 [20]. BA (pH=5.5) was used in this study to simulate the effect of metabolic byproduct anaerobe bacteria [17].

Different methods are used for evaluation of microleakage including dye penetration, bacterial leakage, and fluid filtration [21-25]. Dye penetration and bacterial leakage have been widely used in different studies. These methods are qualitative and destroy the samples, thus they do not allow evaluation of the sealing ability over time. Acidic pH of the environment may discolor the ink used in the dye penetration method. In bacterial leakage method, if the bacteria pass the sample, it may represent positive cultures. In addition, the dry environment used to sterilize the samples may cause cracks in hydrophilic apical plug materials. Thus, the fluid filtration technique introduced by Pashley *et al.* [26] was used in this study to overcome the drawbacks mentioned above. This method has several advantages: it is both quantitative and qualitative; hence it can detect the very small changes. Furthermore, it can be used to evaluate the sealing ability over time since it does not destroy the specimens.

The finding of this study showed that, in comparison with the neutral pH, an acidic environment significantly reduces the sealing ability of MTA and CEM after 30 days. According to previous studies, acidic pH inhibits the setting reactions and increases the solubility of materials. Physical and chemical properties of materials affect their sealing ability [1, 9]. Saghiri *et al.* [16], showed that the leakage of MTA in acidic pH occurs faster than the neutral pH.

On the other hand, the samples in the study by Roy *et al.* [27], were exposed to acidic environment for 24 h, and it was reported that an acid pH significantly reduced the dye leakage of MTA compared with calcium-phosphate cement (CPC).

According to the results of this study, there were not any significant differences between the RML of MTA and CEM cement at both pH levels and all time intervals. This corresponds to the finding presented by Asgary *et al.* [10], who showed no difference between these materials in neutral pH. In this study CEM cement in neutral pH group, exhibited the lowest RML among experimental groups; however, this was not significantly different from MTA. CEM cement has good handling characteristic; it does not adhere to the applicator and thus, could be condensed easily [10]. But Adel *et al.* [5], reported the superior sealing ability of CEM cement over MTA. 

In the current study, the fluid flow rate reduced over time. This is also in agreement with the findings of previous studies which reported the improved sealing ability of MTA [5, 28, 29] and CEM cement [5] with the pass of time.

## Conclusion

Based on the results obtained in this experimental laboratory study, acidic environment reduced the sealing ability of MTA and CEM cement apical plugs. These two endodontic biomaterials exhibited similar sealing ability in acidic pH.
